# On shame and voice-hearing

**DOI:** 10.1136/medhum-2016-011167

**Published:** 2017-04-07

**Authors:** Angela Woods

**Keywords:** Mental health care

## Abstract

Hearing voices in the absence of another speaker—what psychiatry terms an auditory verbal hallucination—is often associated with a wide range of negative emotions. Mainstream clinical research addressing the emotional dimensions of voice-hearing has tended to treat these as self-evident, undifferentiated and so effectively interchangeable. But what happens when a richer, more nuanced understanding of specific emotions is brought to bear on the analysis of distressing voices? This article draws findings from the ‘What is it like to hear voices’ study conducted as part of the interdisciplinary Hearing the Voice project into conversation with philosopher Dan Zahavi's *Self and Other: Exploring Subjectivity, Empathy and Shame* to consider how a focus on shame can open up new questions about the experience of hearing voices. A higher-order emotion of social cognition, shame directs our attention to aspects of voice-hearing which are understudied and elusive, particularly as they concern the status of voices as other and the constitution and conceptualisation of the self.

Hearing a voice in the absence of an external stimulus—what psychiatry terms an auditory hallucination—is an experience which can take many forms and occur in a wide variety of clinical and non-clinical contexts.^1^ The heterogeneity of voice-hearing has given rise to formal taxonomies spanning 16th century theology,^2^ early 20th century psychiatry^3^ ^4^ and 21st century psychology,^5^ ^6^ as well as a myriad of interpretations advanced by individuals using the cultural, medical, spiritual and other hermeneutic frameworks available to them.^7^ ^8^ While varieties of voice-hearing are unlikely to settle into universally accepted subtypes any time soon,^9^ the separation of distressing voices from those that might be regarded as benign or benevolent is made with clarity and consistency. So apparently self-evident is this distinction that the question of what exactly constitutes distress is seldom addressed. Yet, with only a moment's reflection, we can recognise that ‘distress’ could be used to describe voices which are abusive, unrelenting, intrusive, belligerent, hostile, repetitive, violent or overwhelming (in their emotional tone or force and in the semantic content of the utterance); to refer to states of anger, fear, terror, despair, sadness, shame, anxiety, disgust, nausea or exhaustion associated with voice-hearing; and to imply relations of causality, consequence, correlation or coexistence between specific voices and emotions. Clinical and psychological research into the experience of hearing voices has tended to focus on auditory and linguistic processing, and/or the relationship between voices and thoughts; the study of emotions, by contrast, has been relatively neglected. With the importance of attending to the subjective qualities of voice-hearing already recognised in psychotherapeutic^10^ ^11^ and Hearing Voices Movement^8^ ^12^ approaches to working with voices, a fuller examination of the role of the emotions in the temporally dynamic experience of hearing voices is overdue and ripe for interdisciplinary investigation.^13^

This article will focus on shame and voice-hearing, taking as its point of departure the testimony of two voice-hearers gathered by the Hearing the Voice project (http://hearingthevoice.org) in collaboration with the Lived Experience Research Network.^14^ Quotations from the ‘What is it like to hear voices?’ study have been anonymised; demographic information is reported as it was submitted:

Starting when I was about 20 years old, I heard the voices of demons screaming at me, telling me that I was damned, that God hated me, and that I was going to hell. I heard them constantly, even in my sleep. The voices were so frightening and disruptive that much of the time I was unable to focus or concentrate on anything else. […] The voices I heard reflected all the judgmental attitudes I had heard from my family and church about LGBT identities. I internalized that shame and stigma and my own self-loathing brain turned inward and began persecuting itself.

- Shane (white male, queer, late 40s, atheist formerly Catholic, reports having been diagnosed with schizoaffective disorder)

I was under the assumption that the total destruction of my mental faculties was imminent because I had never heard of someone hearing voices and being ok afterwards. I was bewildered and horrified at the foreign sensation and kept thinking I must be making it up and then becoming terrified all over again when the voices persisted in their obtrusiveness. I was very afraid and disoriented and I didn't feel as though I could tell anyone. An immediate sense that this experience was clandestine and something to be ashamed of was present.

- Joelle (white female, bisexual, early 20s, believes in a Judeo-Christian God but not Christianity, reports having been diagnosed with Post Traumatic Stress Disorder)

Shame appears frequently in the first-person accounts of voice-hearers;^8^ ^15^ it is a focus of research investigating the now well-established links between voice-hearing, trauma and childhood sexual abuse;^16–18^ and within a clinical context, cognitive–behavioural and compassion-focused therapies^19–24^ explicitly address and seek to reduce voice-hearers' feelings of shame. Rather than attempt to survey and synthesise these literatures, I want here more modestly to return to first principles and consider how the experience of shame might relate to and reciprocally illuminate some experiences of hearing voices. I say ‘some’ advisedly—in no way do I wish to imply that shame is a meaningful or essential feature of all voice-hearing experiences or that hearing voices is or should be a source of shame. Shame is fascinating because it described so powerfully as being central to people's experience of themselves; it raises questions about temporality, memory, identity and the structure of the self; and it reaches beyond the individual to the other, to their families and communities, past as well as present. By exploring the contention that shame can also help us understand the contents and the structure of some voice-hearing experiences, this article seeks to identify key directions for further research into the nexus between them.

## Psychological and phenomenological perspectives on shame

Shame is increasingly an object of interest across disciplines as diverse as postcolonial literary studies,^25^ neuroscience^26^ and development economics.^27^ Given my focus here on a comparative analysis between the experience of shame and contemporary individuals' experiences of hearing voices, I want to start with social psychological accounts of shame and then go on to show how these are complicated by philosophers working in the existential and phenomenological traditions. While I share the concerns voiced recently by Peter Stearns—that the burgeoning scholarship on shame has largely ignored the historical shift from social *to* psychological shame and that scholars within the social and human sciences have for the most part ‘proceeded on their merry way without much concern for [this] lack of historical ballast’^28^—I hope by the end of this paper to have shown why bringing multiple and historically informed disciplinary perspectives to bear in analysing the experience of shame will be vital to understanding its role in and relationship to voice-hearing.

One of the most widely cited definitions of shame comes from Michael Lewis'^29^ book *Shame: The Exposed Self*. Shame, he writes, is ‘the feeling we have when we evaluate our actions, feelings, or behaviour, and conclude that we have done wrong. It encompasses the *whole of our selves*; it generates a wish to hide, to disappear, or even to die’. Alongside guilt, embarrassment and humiliation, shame has been historically^30^ and cross-culturally^31^ understood as a negative emotion, although one that can serve positive social functions. It is distinguished from so-called basic emotions^32^ such as anger and fear by virtue of its structural complexity: shame is a self-conscious emotion, which requires, or at least implies, a high-order awareness of the self; indeed, it reveals, in Lewis’^29^ formulation, ‘a self exposed to itself’. In this sense, shame is a self-reflective emotion but it is also considered a social one: as well as depending, in a basic sense, on a sophisticated awareness of others' mental states,^33^ the sense of exposure, which, for many, is at the core of shame strongly implies the presence of an interlocutor, whether in the form of specific individuals or society and social norms more broadly. If feelings of guilt often prompt us to seek out others for the purposes of atonement, absolution or reparation, shame by contrast has ‘self-oriented action tendencies’, meaning that it typically results in social withdrawal or retreat.^34^ ^35^ While guilt and shame may share the same stimuli and even co-occur, feelings of guilt pertain to specific actions, behaviours, thoughts or responses, whereas in shame, the entire self, the core of one's subjectivity and identity, is felt to be devalued, diminished or otherwise degraded.^36^ Shame's effects, therefore, can be potentially devastating:

the self is inwardly engaged and preoccupied, paralysed either temporarily or permanently, and unable to engage in taking responsibility and judgement for its own actions; a failed, defiled, unwanted self cannot as a responsive and responsible agent. Perhaps it is not surprising that a shamed person often feels speechless – they fall out of the community of human discourse and responsibility.^37^

Psychology researchers have tended to focus attention on specific moments of shame which are comparatively easy to imagine, identify, recall, compare, and measure. These discrete episodes of ‘acute’ shame, which can help individuals adjust to social norms and expectations, ought to be distinguished from the ‘chronic’ forms of shame, which can come to structure a person's self and situation in often highly negative and damaging ways.^38^ ^39^ Shame can be chronic in a temporal sense, where negative self-evaluations are sustained and intensified through repeated incidences or exposure to a shaming environment. Particularly when examining experiences that are frequently labelled as pathological and aetiologically linked to trauma, it can also be helpful to understand as chronic, in the sense of severe, shame that takes the form of self-stigma. The stigma associated with mental disorders in general, and with the diagnosis of schizophrenia or psychosis specifically, has been extensively analysed^40–43^ and is, for some people, an inextricable part of the distress of hearing voices.^44^

Before pursuing in more depth the relationship between shame and voice-hearing, I want first to turn to the work of the philosopher Dan Zahavi, whose analysis of shame is addressed to two fundamental questions: ‘What does the fact that we feel shame tell us about the nature of self?’ and ‘What kind of self is it that is affected in shame?’^45^ In his critical engagement with the mainstream psychological view of shame that I have just sketched, Zahavi focuses on the role of others and the centrality of self-reflection. Can we feel shame by ourselves, or is the presence of an audience—actual or imagined—essential to the experience? Related to this, is shame best understood as the outcome of an evaluative process, of assessing, comparing and judging the status of the self in relation to our ideals, values and aspirations?

As we have already seen, the experience of shame for Lewis hinges on a process of reflection, analysis and negative self-evaluation: ‘It is not possible’, he writes, ‘to feel shame without comparing one's action against one's standards or beliefs’.^29^ Philosophers Deonna and Teroni place an even greater emphasis on the analytic dimension of shame by defining it as ‘the subject's awareness that the way he is or acts is so much at odds with the values he cares to exemplify that it appears to disqualify him from his very commitment to the value, that is he perceives himself as unable to exemplify it even at a minimal level’.^46^ Zahavi, correctly in my view, cautions against definitions which seem so ‘cognitively demanding’ they pertain only to ‘highly elaborate, self-directed judgemental forms of shame’.^36^ There are, he suggests, more primal and prototypical forms of shame in which it is not reflection but the presence of the other which is critical to and constitutive of the experience.^36^

Here Zahavi turns to Jean Paul Sartre, whose account, in *Being and Nothingness*, emphasises the physiological primacy of shame. Shame is not, first and foremost, the outcome of a deliberative process, but ‘an immediate shudder which runs through me from head to foot without any discursive preparation’.^47^ Sartre continues:

I am ashamed of what I am. Shame therefore realizes an intimate relation of myself to myself. Through shame I have discovered an aspect of my being. Yet although certain complex forms derived from shame can appear on the reflective plane, shame is not originally a phenomenon of reflection. In fact no matter what results one can obtain in solitude by the religious practice of shame, it is in its primary structure shame before somebody.^47^

On this view, the other is not simply a staging post in the process of becoming ashamed, a means to the end of negative self-evaluation. Rather, for Sartre as for Zahavi, the object of one's feeling of shame is the self it is experienced for, through and in the presence of the other. As an emotion which ‘reveals our relationality, our being-for-others’ shame, somewhat unnervingly, ‘makes me aware of not being in control and of having my foundation outside myself’.^36^

For Zahavi, then, it is important to distinguish phenomenologically between a mode of shame which arises from sitting in judgement on ourselves and what he thinks of as the more prototypical experience of shame in the presence of others. Here is a vignette representative of the scenes of ‘disgrace shame’ which are his focus: ‘You have started a new romantic relationship. After a while, in a moment of intimacy, you reveal your sexual preferences. Your disclosure is met by your partner's incredulous stare’.^36^ Zahavi's analysis draws out three features of the phenomenology of shame as it unfolds in this scenario. First, ‘a heightened feeling of exposure and vulnerability’, which is accompanied by the urge to hide, withdraw or disappear and can be arresting to the point of temporary physical incapacity or paralysis. Second, a disruption to the normal temporal flow—rather than being caught up in evaluating past experiences or anticipating future ones, the self is immobilised and immersed in what Karlsson and Sjöberg describe as a ‘frozen now’.^48^ Finally, Zahavi notes that ‘shame, rather than simply involving a global decrease of self-esteem and self-confidence, is also essentially characterized by the way it affects and alters our relationship to and connectedness with others in general’.^36^ In this sense, shame reveals a dimension of our selfhood—the interpersonal self, constituted through the experience and internalisation of the perspective of the other—which bridges the ‘minimal’ self (crudely put, the sense of a first-person perspective) and the more socially, culturally and temporally elaborated narrative self.

## A comparative phenomenology of shame and voice-hearing

Returning to the testimonies of Shane and Joelle, we can see shame clearly at the level of content: Shane is repeatedly ‘damned’ by his voices and told he is evil or unworthy, a judgement that proves inescapable until years later he is able to understand its origins in his community's rejection of his sexual identity; Joelle's experience of ‘obtrusive’ and ‘disorienting’ voices is made doubly frightening by the looping effects of self-stigma—her perception that she is being thrust into the spoiled identity of being a voice-hearer whose mental faculties are by definition destroyed. Via Zahavi's analysis, however, we can also start to see the close and mutually illuminating relationship between shame and voice-hearing at a more structural level. Both Shane and Joelle describe feelings of ‘exposure and vulnerability’: a self-laid bare, an interiority intruded on by alien voices from which there is no escape and no respite. As a continuous and emotionally charged disruption to thinking, articulation and communication, voice-hearing is here portrayed as an overwhelming experience and one in which past and future threaten to collapse into the terrifying reality of the present. Finally, the sense of isolation and disconnection from the social, which for Zahavi and Sartre is one of shame's essential characteristics, is striking: Joelle immediately feels that hearing voices is something she must not disclose and for which she ought to be ashamed; Shane comes to recognise that the voices which disrupted his capacity for social interaction, are also the materialisation of a profound schism between himself and the community in which he grew up.

‘Shame testifies to our exposure, vulnerability and visibility, and is importantly linked to such issues as concealment and disclosure, sociality and alienation, separation and interdependence, difference and connectedness’ writes Zahavi.^36^ The primacy of vision to the experience and conceptualisation of shame is further emphasised by Michael Uebel in his explication of its ethical functions:

Shame is an emotion routed through the eyes and its mise-en-scène is thus specularity and exposure, involving the spatial organization of a spectator who can be external, internal, or both at once. […] Shame is preeminently visual; guilt is aural. […and] Shame necessitates an audience, even when that audience is what is least desired, or struggled against.^49^

Examining an emotion ‘routed through the eyes’ alongside the experience of a voice which cannot be seen alerts us to the ubiquity of metaphor in our efforts to describe inner experience and to the spatial dynamics of self and other. Returning, then, to the key question motivating Zahavi's analysis: what kind of self is it that is affected in shame *and hears voices*? Could certain experiences of hearing voices, such as those we have briefly touched on here, offer an intrapsychic model of the dynamics of shame, to the extent that voices are understood to give a kind of direct perceptual form as well as agency to the perspective and negative judgement of the other? If Zahavi is right that shame discloses something fundamentally important about the role of others not just in the experience but in the *constitution* of the self, what could a more detailed examination of the voice as *self-constituting*-other yield? Does the intensity and persistence of distressing voice-hearing experiences implicate or bear resemblances to forms of shame which are potentially excessive, unrelenting or unresolved? And how might other emotions, moods and affects be involved? In raising these questions, Zahavi's analysis points us towards three major areas of further exploration in the comparative cultural phenomenology of shame and voice-hearing.

### Chronic shame

Zahavi's focus, as we have seen, is on ‘disgrace shame’. The examples he discusses are discrete, episodic exchanges between two or more individuals, which have a physiological immediacy rendering them primary, prototypical and phenomenologically distinct from more reflective forms of shame. Zahavi does not rule out the possibility of such instances having long-lasting effects, but what about cases where shame is experienced repeatedly and systematically; where it is linked to trauma, violence and a wider sense of psychic and physical insecurity; and where it becomes a quality of the familial and social environment itself? Zahavi's analysis, in other words, falls short of investigating what others have called chronic shame, shame that is generated by ‘experiences that induce a sense of persistent inferiority, worthlessness, abandonment, weakness, abjection, unwantedness, violation, defilement, stigmatisation, unloveablility and social exclusion’.^38^ This limitation is not insignificant; as Stephen Pattison notes, there is:

an enormous difference between acute, reactive shame and the chronic shame that shapes a whole personality and may last a lifetime. When individuals appear to experience the whole of life as actually or potentially shame-productive and manifest such symptoms as withdrawal, self-contempt, inferiority, and gaze aversion as a matter of course throughout their everyday lives, shame has become pathological and chronic.^38^

Notwithstanding several very vivid examples of voice-hearing arising in the context of episodes of acute shame,^50^ it is more often the case that voices speak of distress that is better understood as cumulative, structural, enduring or all-encompassing. Indeed, recent research on the relationship between childhood adversity and psychosis has confirmed earlier evidence of a dose–response relationship (ie, that the total number of adverse experiences such as physical or sexual abuse significantly predicts the appearance and severity of voice-hearing), while also showing that ‘two adversities showing the largest number of associations with psychotic symptoms were poverty and being fostered/adopted’.^18^ So while Zahavi's account of shame can help illuminate the ‘now’ of hearing voices (the phenomenology and affective dynamics of the instance in which the voice is heard), experiences of voice-hearing, particularly those that are linked to adversity, might prove particularly fertile for exploring the distinctive phenomenology of chronic shame and for highlighting the importance of sociological analysis to that endeavour.

### Voice as other

The second set of questions Zahavi's account of shame might prompt us to ask about voices concerns their status or ontology: (how) are voices others? For some biomedically oriented hallucinations researchers, the proposition that voices might be best understood in terms of the representation of social agents,^51^ rather than as symptoms of aberrant auditory processing, is already radical. However, within other clinical and psychotherapeutic settings, relational frameworks are routinely used to support voice-hearers to develop more positive relationships with their voices^11^ ^52^ in the way that approaches voices as though they were family members or close acquaintances. Assigning the voice the same status as a person may have practical benefits in a therapeutic context but struggles to account for or attend to the phenomenological heterogeneity of voices. Highly ‘personified’ voices should not be treated as self-evident: for example, while a majority of participants in the ‘What is it like to hear voices?’ study reported that they heard ‘characterful’ voices, one third of respondents indicated that there was nothing person-like about the experience. Of those who did report ‘characterful’ voices, descriptions suggested:

a range of person-like qualities, from amorphous entitativity (an undefined disembodied personality), to stereotypical person-like presentations (an angry man, an old woman), spiritual entities with anthropomorphic traits, specifically recognisable individuals, and voices that are subjectively experienced as representing all or part of the person's own self.^14^

If we are to develop richer accounts of the dynamics of shame and other emotions in voice-hearing experiences, then we must attend more closely to the particular qualities of the agents and entities they most intimately involve. It will also be important not to limit analysis to voices which are bullying or abusive in language and tone, but consider the variety of perhaps more subtle ways in which voices might contribute, positively and negatively, to voice-hearers' experience of shame.

Whatever their ontology, if we nonetheless accept that voices are for a majority of people experienced as other (as being ‘not me’, as something over which the person has little or no control), then the notion of the interpersonal self, as revealed in Zahavi's account of shame, offers new ways of conceptualising the self and other of voice-hearing. Whether psychiatric, psychological, neuroscientific, sociological or spiritual, most frameworks for understanding voices take up one of two positions—either (1) the ‘voice’ is fundamentally independent of the ‘self’ (a disruption in brain activity; the symptom of an underlying biomedical disease; a divine or other-worldly agency) or (2) the ‘voice’ is fundamentally of the ‘self’ (a misrecognised, disowned or dissociated part; a fragment that can be reintegrated into or at least recognised as belonging to the whole). Zahavi's analysis of an interpersonal self which does not exist independently of or prior to the encounter with other, but is in fact *constituted* by it, troubles this dichotomy with the radical proposition that the voice might be *productive of* the self.

### Voices beyond the self

In her powerful analysis of a cultural shift from guilt to shame, Ruth Leys notes that contemporary theorising of shame frequently ‘posits a rigid dichotomy and specular distance between the autonomous subject and the external other’.^53^ Voice-hearing clearly complicates this at every conceivable level, and the work of cultural and affect theorists Grace Cho and Lisa Blackman explicitly implicates shame in the permeability of boundaries between the self and other in voice-hearing. Cho draws on psychoanalytic theories of ‘transgenerational haunting’^54^ to explore the powerful but unspeakable legacies of the Korean War, particularly through the figure of the *yanggongju* (a term used to refer pejoratively to Korean women who have had sex with American men).^55^ The *yanggongju*, embodying a shame which cannot be spoken, haunts the Korean diaspora, posing the question: ‘When the subject cannot speak her own history, when history is unintelligible or made unintelligible, who or what speaks for her?’^55^ Cho invites us to read the hallucinated voices heard by contemporary Korean women not as symptoms of an underlying illness but as the ‘spectral voice of the diasporic unconscious, a voice that has seen things that the hearer has not and that bears witness to the other's past and to the pasts she has inherited.’^55^ The idea that ‘One's mother's voices could be one's grandmother's memories’^55^ shows, as Blackman argues, how voice-hearing experiences:

act on the boundary or threshold between the corporeal and incorporeal, material and immaterial, self and other, psychological and social, past and present, inside and outside, and open our theorizations of affect to the complex forms of mediation which necessarily distribute the psyche beyond a closed, singular, psychological subject.^56^

The work of Blackman and Cho opens up new ways of conceptualising the self and other of voice-hearing as testifying to the interpersonal and intergenerational dynamics of shame. In so doing, it also suggests that phenomenology is not simply coloured by but more fundamentally constituted through a network of social relations which cannot be abstracted from wider logics of race, class, gender, sexuality and history.

## Conclusion: directions for further research

The literatures on shame and on voice-hearing are extensive, interdisciplinary and deserving of a deeper analysis than has been possible here. Rather than seek to reconcile, synthesise or arbitrate between them, I hope instead to have shown that the intersections between these literatures demand analysis that is alive to competing models of shame (from social psychology, phenomenology, cultural theory and the history of emotion), to a variety of ways of understanding the phenomenology and aetiology of hearing voices, and to the potential for there to be complex interactions between them at the level of testimony as well as theorisation. In its emphasis on the presence of other, Zahavi's discussion of shame is, I have argued, particularly helpful in analysing the way self and other are conceptualised and constituted in experiences of voice-hearing. Specifically, Zahavi's notion of the interpersonal and inherently other-constituted self might better equip us to attend to the complexity of voice-hearers' self-constituting relationships with the other of their voices, which in turn could help nuance the models of shame that inform relational, compassionate mind and cognitive–behavioural therapy approaches. Finally, and with the testimony of Shane and Joelle firmly in mind, this analysis has shown that we need to understand and account for shame at multiple levels: to grasp how the shaming experience of abusive voices that no one else can hear relates to the public stigma of being a voice-hearer; how homophobia can manifest in and as an internal demonic drama and how voices might bear the traces of trauma experienced collectively as well as individually. A robust and critical medical humanities approach^13^ ^57^ to these issues will call on a wide range of disciplinary and clinical expertise and, crucially, ensure that people who hear voices, and for whom shame is an intimate and painful aspect of experience, are at the forefront of future investigations.
